# Olecranon Fractures: Patterns, Fixation Types, and Outcomes

**DOI:** 10.7759/cureus.106381

**Published:** 2026-04-03

**Authors:** Kashif Memon, Raghunaathan Rangan, Musab Mohamed, Ariz Raza, Ross Fawdington, Edward Spurrier

**Affiliations:** 1 Trauma and Orthopedics, Queen Elizabeth Hospital Birmingham, Birmingham, GBR

**Keywords:** hardware irritation, olecranon fracture, orthopedic trauma, plate fixation, prominent metalwork, suture fixation, tension band wiring

## Abstract

Background: Prominent metalwork remains a common source of postoperative discomfort following fixation of olecranon fractures and often leads to elective implant removal despite successful union. This study aimed to assess the incidence and pattern of symptomatic hardware prominence among different fixation methods involving tension band wiring (TBW), plate fixation, and suture constructs in a tertiary trauma center.

Methods: A retrospective review was conducted of adult patients who underwent operative fixation for olecranon fractures at the Queen Elizabeth Hospital, Birmingham, between January 2021 and July 2025. Patients with a minimum follow-up of 12 weeks were included. Both open and closed fractures were analyzed. Fixation methods included TBW, plate fixation using pre-contoured or hook plates, and high-strength fiber-suture constructs. The primary outcome was the reoperation rate, along with other fixation-related complications. Data were analyzed using Fisher’s exact test, with p<0.05 considered statistically significant.

Results: Of the 95 patients, 62 met the inclusion criteria (30 males, 32 females; mean age 57 years). The mean follow-up was 46 weeks (range, 3-42 months). TBW was used in 11 cases, plate fixation in 46, and suture fixation in five. A total of 22 patients experienced complications, of which 15 were due to prominent metalwork. Other complications requiring surgery included nonunion (1), synostosis (1), muscle hernia (1), and revision of radial head replacement (1). Three patients with loss of reduction did not undergo further surgery due to subsequent functional improvement. Sixteen patients had open fractures, with no postoperative infections noted.

Of the 15 patients with prominent metalwork, 11 underwent implant removal (three in the TBW group and eight in the plating group). There was a difference in the incidence of removal of symptomatic metalwork between TBW (3/11, 27%) and plating (8/46, 17%), although the overall rate of symptomatic metalwork was similar in both groups (27% vs. 26%, respectively).

Conclusion: While both TBW and plate fixation provide stable fixation and reliable union, prominent metalwork remains a frequent postoperative issue and the primary reason for secondary surgery. Careful implant selection, meticulous soft‑tissue handling, and patient counseling are essential to minimize postoperative discomfort and optimize outcomes.

## Introduction

Olecranon fractures represent approximately 10% of all upper limb fractures and most commonly occur following direct trauma or falls onto the elbow [1]. Management aims to restore the articular surface, maintain stable fixation, and allow early motion to prevent stiffness. Treatment strategy is primarily guided by the Mayo classification, first described by Cabanela and Morrey in 1993, which stratifies injuries according to displacement and stability [1-3]. There is a broad consensus that Mayo type 1A and 1B fractures are nondisplaced and should be treated non-operatively with immobilization and early range-of-motion exercises [1].

The optimal fixation technique for displaced but stable fractures, particularly Mayo type 2A, remains a subject of ongoing debate [4]. Tension band wiring (TBW) has traditionally been the mainstay of treatment for simple transverse olecranon fractures, providing stable fixation through dynamic compression. However, it is increasingly associated with symptomatic hardware and metalwork prominence due to the subcutaneous position of the olecranon, leading to irritation, discomfort, and a high rate of elective implant removal [5-7]. Plate fixation provides rigid stability and may better resist triceps pull in comminuted or osteoporotic bone, but can similarly cause discomfort and prominence due to limited soft-tissue coverage, despite advances in low-profile designs [8-10].

Several recent studies, including the randomized controlled trial by Duckworth et al. [5] and a meta-analysis by Mikhail et al. [6], have reported comparable functional outcomes between TBW and plate fixation but consistently higher rates of hardware-related irritation following TBW. For more complex fractures (Mayo types 2B, 3A, and 3B), plate fixation remains the preferred method, supported by studies such as Bailey et al. [7] and Erturer et al. [8], which demonstrated satisfactory union but noted symptomatic implant prominence in 9% to 56% of patients.

Suture-based fixation using high-strength fiber materials has recently emerged as a viable alternative, particularly for simple transverse fractures. Das et al. [9] reported excellent outcomes and complete union in all patients treated with suture fixation, with no hardware irritation or reoperations. This technique offers adequate stability while avoiding the soft-tissue irritation commonly associated with metallic implants [10].

Given the ongoing challenge of implant prominence and hardware-related discomfort following olecranon fracture fixation, this study aimed to evaluate the incidence and patterns of prominent metalwork among different fixation techniques, including TBW, plate fixation, and suture constructs, at a single tertiary trauma center over a five-year period.

The aim of this study was to evaluate the incidence and patterns of symptomatic hardware prominence following operative fixation of olecranon fractures using TBW, plate fixation, and suture-based constructs. The primary outcome was the occurrence of symptomatic hardware prominence. Secondary outcomes included the overall reoperation rate and other postoperative complications, including nonunion, synostosis, implant failure, and soft-tissue irritation. In addition, outcomes associated with suture fixation were assessed to determine its viability as an alternative technique in selected fracture patterns.

## Materials and methods

This retrospective observational study was conducted at the Queen Elizabeth Hospital Birmingham, a tertiary referral center, between January 2021 and July 2025. All adult patients who underwent operative fixation for olecranon fractures during this period were identified retrospectively from theatre records and electronic clinical databases, encompassing both open and closed injuries. Data were obtained from operative notes, clinic records, and radiographic imaging. As the project constituted a service evaluation using anonymized data, formal ethical approval was not required. However, clinical audit registration and management approval were obtained (approval number 23606).

Inclusion and exclusion criteria

Eligible participants included adult patients aged 18 years or older who underwent operative fixation for an olecranon fracture at our tertiary referral center between January 2021 and July 2025. Patients were required to have a minimum postoperative follow-up duration of 12 weeks to allow adequate assessment of fracture healing and complications. Both open and closed fractures were considered for inclusion.

Patients with Mayo type I fractures were excluded, as these injuries were managed non-operatively at our institution. Individuals with incomplete clinical documentation or less than 12 weeks of follow-up were also excluded. Additionally, patients with associated ipsilateral elbow injuries requiring separate fixation procedures, such as distal humeral fractures, were excluded to avoid confounding the analysis of outcomes related specifically to olecranon fracture fixation.

A total of 95 patients were initially identified. Following application of the inclusion and exclusion criteria, 62 patients were included in the final analysis.

Fractures were classified using the Mayo classification system, originally described by Cabanela and Morrey (1993) and later reviewed by Sullivan and Desai (2019) [2,3]. This system categorizes injuries according to displacement, stability, and comminution, as outlined in Table 1.

**Table 1:** Mayo classification of olecranon fractures Adapted from references [2,3].

Type	Description
I	Undisplaced fracture
IIA	Displaced, stable, non-comminuted fracture
IIB	Displaced, stable, comminuted fracture
IIIA	Displaced, unstable, non-comminuted fracture
IIIB	Displaced, unstable, comminuted fracture

Demographic information, fracture classification, fixation method, and postoperative outcomes were recorded. The fixation technique was selected based on fracture morphology, stability, degree of comminution, and bone quality. TBW was utilized mainly for simple transverse fractures with adequate bone stock. Plate fixation was chosen for comminuted, osteoporotic, or unstable injuries, particularly where greater stability was required to counteract triceps forces or where there was articular disruption. High-strength suture fixation was used selectively for simple type IIA fractures or minimal comminution in which stable reduction could be achieved without metal implants. The final decision regarding fixation method was made intraoperatively by the treating surgeon based on these factors.

All procedures were performed under general anesthesia. Patients were positioned either supine or lateral, with the operative limb supported across the chest or on a radiolucent armrest. A posterior approach was used in all cases to achieve direct exposure and anatomic reduction. TBW was undertaken using the standard Arbeitsgemeinschaft für Osteosynthesefragen (AO) technique with parallel Kirschner wires and stainless-steel wire loops. Plate fixation involved pre-contoured olecranon plates (Synthes, Oberdorf, Switzerland; Smith & Nephew, Watford, United Kingdom), secured with a combination of locking and cortical screws. Three proximal avulsion fractures were managed with a two-hole hook plate (Synthes, Oberdorf, Switzerland), and one case with a plate incorporating tines (Smith & Nephew, Watford, United Kingdom).

Kirschner wires and stainless-steel cerclage wire used for TBW were standard AO instrumentation (AO Foundation, Davos, Switzerland). Suture fixation was performed using No. 2 FiberWire in a combined figure-of-eight and cerclage configuration, with reinforcement using Krakow FiberWire sutures through the triceps, passed through a transverse olecranon drill hole to de-tension the fracture site, thereby promoting healing and preventing displacement. Postoperative wound checks were performed at two weeks, with radiographs obtained at six and 12 weeks. Patients were encouraged to commence early active elbow motion as tolerated. Individuals with delayed healing, complex injuries, or postoperative complications were followed for longer durations.

The primary outcome was the incidence of symptomatic hardware prominence, defined as painful hardware causing discomfort when resting or applying pressure on the posterior elbow, and was assessed clinically during follow-up based on patient-reported symptoms and examination findings. Secondary outcomes included the overall reoperation rate and other postoperative complications, including nonunion, synostosis, implant failure, and soft-tissue irritation. Reoperations were defined as any secondary surgical procedure related to the index fixation.

All postoperative radiographs were independently reviewed by two orthopedic surgeons to confirm fracture union and assess implant position, prominence, and mechanical integrity. Fracture union was defined as the presence of bridging callus across the fracture site with no evidence of implant failure or progressive displacement. Loss of reduction was defined as a change in fracture alignment compared to immediate postoperative imaging. In cases of disagreement, a consensus decision was reached.

Statistical analysis

Statistical analysis was performed using Fisher’s exact test to evaluate associations between fixation method, fracture classification, and complication rates. This test was selected due to the relatively small sample size and the categorical nature of the variables. Continuous variables were summarized using means and ranges, while categorical variables were presented as frequencies and percentages. Statistical significance was defined as a p-value less than 0.05. Data analysis was conducted using IBM SPSS Statistics (IBM Corp., Armonk, New York, USA). No formal sample size calculation or power analysis was performed due to the retrospective nature of the study.

## Results

A total of 62 patients met the inclusion criteria after excluding those with less than 12 weeks of follow-up. The cohort consisted of 30 males and 32 females, with ages ranging from 19 to 93 years (mean, 57 years). The mean follow-up duration was 10 months (range, 3-42 months). Sixteen patients had open fractures, and both open and closed injuries were included in the analysis (Table 2).

**Table 2:** Demographic and clinical characteristics of the study cohort (n=62)

Variable	Category	Number	Percentage
Gender	Male	30	48.40%
Gender	Female	32	51.60%
Age (years)	Mean (range)	57 (19-93)	-
Fracture type	Open	16	25.80%
Fracture type	Closed	46	74.20%
Follow-up duration	Mean (range)	10 months (3-42)	-

According to the Mayo classification, 10 fractures were type IIA, 20 were type IIB, six were type IIIA, and 26 were type IIIB. Fixation methods included TBW in 11 patients, plate fixation in 46 patients, and suture fixation in five patients. The distribution of fracture types, fixation techniques, and corresponding reoperation rates is summarized in Table 3.

**Table 3:** Distribution of fracture type, fixation method, and reoperation rates TBW: tension band wiring

Mayo type	TBW, n (%)	Plate, n (%)	Suture, n (%)	Total, n (%)	Reoperations, n (%)
IIA	3 (30%)	4 (40%)	3 (30%)	10 (100%)	2 (20%)
IIB	4 (20%)	14 (70%)	2 (10%)	20 (100%)	5 (25%)
IIIA	1 (17%)	5 (83%)	0 (0%)	6 (100%)	1 (17%)
IIIB	3 (12%)	23 (88%)	0 (0%)	26 (100%)	7 (27%)
Total	11 (18%)	46 (74%)	5 (8%)	62 (100%)	15 (24%)

Symptomatic metalwork was the most frequent complication and the leading indication for reoperation (11 of 15 cases), as outlined in Table 4 and further detailed by fixation method in Table 5. Additional reasons for secondary procedures included one nonunion requiring revision, one post-traumatic synostosis excision, one peri-implant fracture fixation, and one radial head revision, all of which involved concurrent removal of olecranon plate fixation (Table 5). These additional reoperations are summarized in Table 6.

**Table 4:** Symptomatic metalwork and implant removal by fixation method TBW: tension band wiring

Fixation method	Total patients	Symptomatic metalwork, n (%)	Implant removal for prominence, n (%)	Symptomatic managed non-operatively, n (%)
TBW	11	3 (27%)	3 (27%)	0 (0%)
Plate fixation	46	12 (26%)	8 (17%)	4 (9%)
Suture fixation	5	0 (0%)	0 (0%)	0 (0%)
Total	62	15 (24%)	11 (18%)	4 (6%)

**Table 5:** Comparison of complications between plate, TBW, and suture fixation TBW: tension band wiring

Complication type	Plate (n=46)	TBW (n=11)	Suture (n=5)
Prominent metalwork	12 (26%)	3 (27%)	0 (0%)
Surgery for prominence	8 (17%)	3 (27%)	0 (0%)
Surgery for nonunion	1 (2%)	0 (0%)	0 (0%)
Surgery for synostosis	1 (2%)	0 (0%)	0 (0%)
Surgery for radial head revision	1 (2%)	0 (0%)	0 (0%)
Surgery for peri-implant fracture	1 (2%)	0 (0%)	0 (0%)
Metal failure, no surgery	3 (7%)	0 (0%)	0 (0%)
Prominence, no surgery	4 (9%)	0 (0%)	0 (0%)
Muscle hernia, no surgery	1 (2%)	0 (0%)	0 (0%)

**Table 6:** Reoperations for non-prominence-related indications

Indication	Number of cases, n (%)
Nonunion requiring revision	1 (2%)
Post-traumatic synostosis excision	1 (2%)
Peri-implant fracture fixation	1 (2%)
Radial head revision	1 (2%)
Total	4 (6%)

Fifteen patients developed clinically significant metalwork prominence following TBW or plate fixation. In the TBW group, three of 11 patients (27%) underwent K-wire removal due to pain or irritation after confirmed union. In the plate fixation group, 12 of 46 patients (26%) reported prominent metalwork; however, only eight (17%) elected to undergo implant removal, while four opted for non-operative management. Four additional plate-treated patients required reoperation for non-prominence-related reasons, as listed in Table 5. Four patients were treated with hook plates, all of which achieved union; two reported mild discomfort when resting the elbow on hard surfaces. No hardware-related issues occurred in the five suture fixation cases (Table 7).

**Table 7:** Outcomes of hook plate and suture fixation

Fixation type	Number of patients	Union achieved	Symptomatic prominence	Reoperation required
Hook plate fixation	4	4 (100%)	2 (50%) mild symptoms	0 (0%)
Suture (FibreWire) fixation	5	5 (100%)	0 (0%)	0 (0%)

The overall complication rate was 35% (22 of 62 patients). The overall reoperation rate was 24% (15 of 62 patients), with 73% (11 of 15) performed for symptomatic hardware prominence (Table 8). Among those treated with plates, five additional complications did not require surgery: three cases of loss of reduction (all with good functional recovery), one muscle hernia, and two cases of asymptomatic plate prominence. No infections were observed in this study, and all fractures achieved radiologic union except for one case of nonunion after plating. The overall complication profile is summarized in Table 9.

**Table 8:** Summary of reoperation indications

Outcome measure	Number of patients, n (%)
Total reoperations	15 (24%)
Reoperations for symptomatic prominence	11 (73%)
Reoperations for other causes	4 (27%)

**Table 9:** Overall postoperative complications

Complication	Number of patients, n (%)
Symptomatic hardware prominence	15 (24%)
Loss of reduction (no surgery)	3 (5%)
Muscle hernia	1 (2%)
Asymptomatic plate prominence	2 (3%)
Nonunion	1 (2%)
Synostosis	1 (2%)
Peri-implant fracture	1 (2%)
Radial head revision	1 (2%)
Total complications	22 (35%)

Plate fixation outcomes were further analyzed according to the Mayo classification (Table 10). Type IIA fractures showed the highest proportion of reoperations, although the numbers were too small to draw firm conclusions. Notably, five patients treated with suture (FiberWire) fixation for type IIA/IIB fractures experienced no clinical symptoms. Although early mild separation was noted on X-rays, it had no impact on outcomes and did not require reoperation.

**Table 10:** Plate fixation data based on fracture types

Type	Number of plate fixations n (%)	Number of reoperations n (%)	Reoperation rate (%)
IIA	4 (8.7%)	2 (4.3%)	50%
IIB	14 (30.4%)	4 (8.7%)	29%
IIIA	5 (10.9%)	1 (2.2%)	20%
IIIB	23 (50.0%)	5 (10.9%)	22

Comparison of fixation methods showed no statistically significant difference in reoperation rates between the plate, TBW, and suture groups (p=0.42). Reoperations for symptomatic metalwork prominence occurred in three of 11 TBW patients (27%) and eight of 46 plate fixation patients (17%). Fisher’s exact test showed no significant difference between groups (p=0.43). All suture fixation cases remained free of prominence or symptomatic sutures throughout follow-up.

## Discussion

In this retrospective analysis, we examined the incidence of prominent metalwork and associated complications following operative fixation of olecranon fractures using TBW, plate fixation, and suture-based constructs. As all Mayo type IA and IB fractures were treated non-operatively and therefore excluded, the study focused exclusively on displaced injuries requiring surgical stabilization. The findings of this study are consistent with previous literature demonstrating that, although both TBW and plating reliably achieve fracture union, postoperative hardware prominence remains a common and clinically significant problem.

In our cohort, symptomatic metalwork prominence necessitating removal occurred in 27% of TBW cases and 17% of plate fixations. This is consistent with the randomized controlled trial by Duckworth et al. [5], which reported that the majority of reoperations were prompted by hardware irritation rather than mechanical complications, with reoperation rates of 63% in the TBW group and 38% following plate fixation. Similar trends were identified in the 2025 meta-analysis by Mikhail et al. [6], which concluded that TBW was associated with a significantly higher rate of hardware-related complications, despite comparable functional outcomes. Giardina et al. [11] also observed that TBW resulted in more frequent reoperations, whereas plating, although requiring longer operative times, was associated with fewer symptomatic cases and no increase in hospital stay. However, the relatively small and uneven sample sizes across fixation groups in the present study limit the ability to draw definitive comparative conclusions between techniques.

Further corroboration comes from the systematic review by Wang and Li [12], which demonstrated hardware-related complication rates of 30% for TBW and 16% for plate fixation, with reoperation rates of 15% and 9%, respectively. These data reinforce the concept that modern plating systems provide more predictable fixation with fewer postoperative issues. The anatomical characteristics of the olecranon, particularly its subcutaneous location and limited soft-tissue envelope, contribute to the high risk of symptomatic implant prominence once postoperative swelling subsides. Symptomatic hardware can cause discomfort during rest or when leaning on the elbow, prompting elective removal even after successful union. Although low-profile and anatomic plates have been developed to mitigate irritation, symptomatic metalwork prominence remains a recognized disadvantage of fixation, with removal rates increasing over time [7,8,11-13].

In the plating cohort of this study, symptomatic prominence occurred in 26% (12 of 46 cases), but removal occurred in 17% (8 of 46 cases), based on patient choice, affecting both dorsal and hook plates. These findings align with Bailey et al. [7], who reported plate removal in 20% of patients due to posterolateral irritation, and Erturer et al. [8], who noted high union rates with plate fixation but also frequent complaints of implant prominence. The use of modern low-profile implants, particularly those with pre-contoured designs, may reduce soft-tissue irritation without compromising stability [11]. Several authors [12,14] have also advocated plate fixation with supplementary suture augmentation to counteract triceps tension in osteoporotic bone or avulsion-type fractures. Bosman et al. [15] demonstrated that intramedullary screw fixation can provide adequate stability while avoiding prominent posterior hardware and may therefore reduce the risk of symptomatic implant-related complications.

Suture fixation using high-strength fiber materials represents an alternative strategy that eliminates metal hardware and the associated risk of symptomatic prominence, although it may not be suitable for all fracture configurations, and loss of anatomic reduction can occur without meticulous technique. In the current series, although used in only five patients, suture constructs were not associated with symptomatic irritation or reoperation; however, the very small sample size limits the strength of this observation and precludes definitive conclusions regarding efficacy. These findings echo those of Das et al. [9], who reported full union and no reoperations in ten patients at a mean follow-up of 19 months. Such constructs are particularly attractive for simple, non-comminuted or minimally comminuted fractures where stable reduction and dynamic compression can be achieved without implants, offering a viable alternative for selected cases [10].

Economic considerations also play a role in implant selection, although the literature presents mixed conclusions once reoperation rates and hardware removal are factored in. Steadman et al. [16] identified TBW as the most cost-effective method for Mayo type IIA fractures, even after accounting for secondary procedures. Francis et al. [17] reached similar conclusions, finding that TBW remained cost-effective when reoperation rates and plate costs were considered. However, the clinical burden of patient discomfort, combined with the use of additional operating room resources for elective hardware removal, suggests that reducing the incidence of symptomatic metalwork may have both clinical and economic advantages over the longer term.

Overall, this study reinforces that symptomatic hardware prominence, rather than fixation failure, is the primary cause of postoperative symptoms and reoperation following olecranon fracture fixation. Both TBW and plate fixation were associated with notable rates of symptomatic metalwork irritation (27% and 26%, respectively), whereas low-profile plates and suture-based constructs may offer ways to reduce prominence-related complications. Appropriate preoperative counseling and careful implant selection remain essential in setting patient expectations and optimizing satisfaction.

In summary, although both TBW and plating provided reliable fracture union, prominent metalwork was a frequent postoperative issue, and most secondary procedures were elective, undertaken after fracture healing due to discomfort when resting the elbow. While these findings suggest potential advantages of low-profile plates and suture-based techniques, any comparative interpretation between fixation methods is limited by the small and uneven sample sizes and should be considered descriptive rather than definitive, and requires validation in larger, prospective, controlled studies.

The main limitations of this study include its retrospective design, relatively small sample size, and variability in fixation techniques. The absence of a formal power calculation is also recognized as a limitation. Additionally, functional outcome measures, such as range of motion and patient-reported scores, were not consistently documented. Furthermore, the non-randomized selection of fixation technique introduces potential selection bias, as treatment decisions were influenced by fracture characteristics and surgeon preference, which were not controlled for in the analysis. The lack of validated functional outcome measures, such as MEPS or DASH scores, limits the assessment of the clinical impact of complications on patient function. These factors limit the ability to perform robust comparative analysis between fixation methods and reduce the generalizability of the findings. Despite these limitations, the study provides meaningful real-world data regarding symptomatic implant prominence patterns and their implications for improving future clinical practice.

## Conclusions

Both TBW and plate fixation remain effective for achieving union in olecranon fractures; however, prominent metalwork is a frequent source of postoperative discomfort and the primary reason for secondary procedures. Similar rates of symptomatic hardware irritation were observed between TBW and plate fixation; however, the study was not powered to detect significant differences between groups. Low-profile plates may reduce, but not eliminate, this issue. Suture fixation was not associated with symptomatic hardware in this small cohort; however, the limited sample size precludes definitive conclusions regarding its effectiveness or broader applicability. Careful implant selection, meticulous soft-tissue handling, and preoperative counseling regarding the risk of hardware irritation are essential to optimize patient satisfaction and reduce reoperation rates.
